# The relationship between thyroid function and ovarian reserve: a prospective cross-sectional study

**DOI:** 10.1186/s13044-021-00112-2

**Published:** 2021-10-01

**Authors:** Roya Kabodmehri, Seyedeh Hajar Sharami, Ziba Zahiri Sorouri, Nasrin Ghanami Gashti, Forozan Milani, Zeinab Chaypaz, Maryam Ghalandari

**Affiliations:** 1 Reproductive Health Research Center, Department of Obstetrics & Gynecology, Al-Zahra Hospital, School of Medicine, Guilan University of Medical Sciences, Namjoo Street, P.O.Box: 4144654839, Rasht, Iran; 2 Mehr Fertility Research Center, Guilan University of Medical Sciences, Rasht, Iran; 3 Vice-Chancellorship of Research and Technology, Guilan University of Medical Science, Rasht, Iran

**Keywords:** Thyroid dysfunction, ovarian reserve, TSH, AMH

## Abstract

**Background:**

Thyroid dysfunction can affect fertility and miscarriage risk by affecting the process of follicular growth, embryo development, implantation, and placental formation. It has been suggested that thyroid disorders are associated with ovarian reserve by affecting the follicular process. The aim of the present study was to investigate the relationship between thyroid hormone levels and ovarian reserve.

**Methods:**

Three hundred fourteen women with infertility due to various etiologies were enrolled in this study (172 individuals with Anti-Mullerian hormone (AMH) level ≥ 1.1 ng/ml and 142 individuals with AMH < 1.1 ng/ml). Serum levels of follicle-stimulating hormone (FSH), estradiol (E2) on day 2–4 of menstrual cycles, AMH, Thyroid-stimulating hormone (TSH), and thyroxine (free T4) were evaluated.

**Results:**

In participants with age over 35 years, median TSH level in women with AMH < 1.1 ng/ml was significantly higher than those with AMH ≥1.1 ng/ml (*P*-value =0.037). There was no significant difference in body mass index (BMI) in patients with age older than 35 years and younger than 35 years sub-groups based on AMH level (*P*-value = 0.102, and *P*-value = 0.909 respectively). With one unit increase in TSH level, the odds of having AMH < 1.1 ng/ml increases by 1.25 times or by 25% (*P*-value =0.017). Receiver operator characteristic (ROC) curve analysis showed a TSH cut-off point of 1.465 mIU/L in participants over 35 years in identifying decreased AMH level.

**Conclusion:**

Our study supports the relationship between TSH level and ovarian reserve so that with an increase in TSH from a certain level is associated with a decrease in ovarian function.

## Background

Decreased ovarian reserve and subsequent reduction in the quantity and quality of oocytes is a process that depends on age. However, this phenomenon can occur at any age. In younger patients, it is mostly idiopathic but there are other causes such as ovarian surgery, endometriosis, and chemo-radiotherapy in reproductive age. Anti-Mullerian hormone (AMH) is a glycoprotein produced from granulosa cells of follicles, sized between of 5 to 8 mm [1], and is a good biomarker for ovarian reserve. Its level is constant throughout the menstrual cycle and has a high specificity for detecting the inadequate ovarian response [2].

One of the factors affecting the functional reserve of the ovary is thyroid disorders. Thyroid diseases are one of the most common endocrine problems in reproductive age and may cause menstrual and ovulation disorders and infertility. The prevalence of subclinical and clinical hypothyroidism in women of reproductive age is 4–10 and 0.1–2%, respectively [3]. The exact mechanism of the effect of thyroid disorders on menstruation and infertility is not precisely understood. Oocytes are surrounded by granulosa cells that are evolving as vital support for oocytes. Studies show that different cells in the ovary, including oocytes, granulosa cells, and epithelium express receptors of thyroid hormone. TSH has a synergistic effect with follicle stimulating hormone (FSH) in promoting the proliferation of granulosa cells [4]. On the other hand, studies have shown that thyroid hormones regulate FSH stimulation in follicles and prevent their apoptosis [5]. In the process of folliculogenesis, thyroid hormones bound to thyroid-binding globulin (TBG) increases with increasing serum estrogen levels, and the result of these phenomena is a decrease in free thyroxin and an increase in TSH level [6]. Thyroid hormone is also found in follicular fluid. It plays an important role in the process of follicle development [5] and its dysregulation may impair follicular development.

Thyroid hormone also plays an important role in the process of implantation and early development of the fetus through its effect on the endometrium and placenta, which can be used to regulate the invasion of the extra villus trophoblast by affecting the matrix metalloproteinase [7, 8]. The expression of thyroid hormone receptors in the ovary and endometrium and its important role in endometrial receptivity, implantation window, placenta, and embryonic tissues has shown [4]. Studies examining the relationship between thyroid hormone level and ovarian reserve have shown conflicting results [9, 10]. A number of studies support the association between high serum TSH level and decreased ovarian reserve; also, in euthyroid patients, TSH below 3 mIU/ml has been shown to be associated with better ovarian reserves [11, 12]. On the other hand, this association has not been confirmed by other studies [13]. With increasing age, both ovarian reserve decreases, and thyroid disorders increase, therefor it is possible that there is an association between these two conditions. The aim of the present study was to investigate the relationship between thyroid hormones levels and ovarian reserve.

## Methods

The present study is a prospective cross-sectional study that was conducted between 2019 and 2020 in Al-Zahra Hospital after obtaining approval from the Ethics Committee of Guilan University of Medical Sciences (Approval ID: IR.GUMS.REC.1399.541). A total of 314 women with infertility due to various causes (172 individuals with AMH ≥1.1 ng/ml and 142 individuals with AMH < 1.1 ng/ml) were included in the study. All women with infertility (not being able to get pregnant after 1 year or longer of unprotected sex) referred to the infertility clinic of Al-Zahra hospital, Rasht, Iran were included in the study. Exclusion criteria were any iatrogenic cause for decreased ovarian reserve such as ovarian surgery, radiotherapy and chemotherapy, endometriosis, history of known thyroid disorders, levothyroxine users, polycystic ovarian syndrome (PCOS), and patients who declined to participate in the study. Informed consent was obtained from all patients before enrollment. All participants had normal serum prolactin levels. The levels of FSH and estradiol (E2) on days 2–4 of the menstrual cycle (Siemens Immulite 2000 xpi Immunoassay System, Germany), AMH (Immunotech Beckman coulter ELISA kit, USA), TSH, and free T4 (Architect Abbott i 2000 SR, USA) were measured. The researcher-made checklist included demographic (age, BMI) and hormonal levels (FSH, luteinizing hormone (LH), AMH, TSH, Free T4, and E2) were recorded. The manufacturer’s reference ranges were 0.45–4.5 mIU/L for TSH, and 12–22 pmol/L for free T4. Subclinical hypothyroidism was defined as TSH > 4.5 mIU/mL with the free T4 concentration within the reference range. Patients with TSH > 4.5 mIU/L and low free T4 were considered as affected by clinical hypothyroidism.

### Statistical analysis

The data were analyzed by using the IBM SPSS software (v 21.0. Armonk, NY, USA). The normal distribution of quantitative variables was assessed using the Kolmogorov–Smirnov test. The median and the interquartile range (IQR) were used for analyzing quantitative variables, and numbers and percentages were used for qualitative variables. The relationship between clinical hypothyroidism and subclinical hypothyroidism variables in both AMH groups was analyzed using the Chi-square test and Fisher’s exact test. Comparison of hormonal variables in the two groups of participants in the study (AMH ≥1.1 ng/ml and AMH < 1.1 ng/ml) was analyzed using Mann–Whitney U test. The relationship between AMH and Free T4, FSH, TSH, and E2 was analyzed using the partial correlation test. Then, variables that had a *P*-value of less than 0.2 in the partial correlation test were selected and were analyzed by multiple logistic regression analysis. In addition, we used the receiver operator characteristic (ROC) curve to estimate the cut-off point of TSH in identifying decreased AMH level. The statistical significance level was considered at *P*-value < 0.05.

## Results

A total of 314 women with infertility due to various causes (172 individuals with AMH ≥1.1 ng/ml and 142 individuals with AMH > 1.1 ng/ml) were enrolled in this study. Mean age of participants was 36.66 ± 6.12 years. They were divided into two groups: patients with age 35 years or older and younger than 35 years. There was no significant difference in BMI in patients with age older than 35 years and younger than 35 years sub-groups based on AMH level (*P*-value = 0.102, and *P*-value = 0.909 respectively) (Table 1). The number of participants younger than 35 years was 124 (49.39%) while the number of participants aged 35 years or older was 190 (51.60%). Seventy percent of individuals aged 35 years or over and 19.4% of individuals younger than 35 years had AMH < 1.1 ng/ml (*P*-value =0.001). Participants were evaluated for TSH, Free T4, FSH, E2 variables in each age group (Table 1). In participants with age over 35 years, median TSH level in women with AMH < 1.1 ng/ml was significantly higher than those with AMH ≥1.1 ng/ml (*P*-value =0.037). Also, FSH level was significantly higher in individuals with AMH < 1.1 ng/ml as compared to those with AMH ≥1.1 ng/ml in both the age groups (*P*-value = 0.001). Participants with AMH < 1.1 ng/ml and those with AMH ≥1.1 ng/ml had similar levels of E2 and Free T4 in both age groups (*p* > 0.05) (Table 1).

**Table 1:** Clinical characteristics and hormones level in the two sub-groups of participants based on Anti-Mullerian hormone (AMH) levels.

		Age ≤ 35 years			Age > 35 years		
		AMH < 1.1 ng/ml Median (IQR)	AMH ≥ 1.1 ng/ml Median (IQR)	*P*-value	AMH < 1.1 ng/ml Median (IQR)	AMH ≥ 1.1 ng/ml Median (IQR)	*P-value
BMI		26.6 (24.96–29.2)	27.0 (24.50–29.77)	0.909	27.6 (25.5–30.4)	28.4 (26.68–31.32)	0.102
Clinical hypothyroidism	yes	2 (66.7%)	1 (33.3%)	0.096	6 (100%)	0	0.181
	no	22 (18.2%)	99 (81.8%)		127 (69%)	57 (31%)	
	total	24 (19.4%)	100 (80.6%)		133 (70%)	57 (30%)	
Subclinical hypothyroidism	yes	2 (28.6%)	5 (71.4%)	0.525	15 (83.3%)	3 (16.7%)	0.194
	no	22 (18.8%)	95 (81.2%)		118 (68.6%)	54 (31.4%)	
	total	24 (19.4%)	100 (80.6%)		133 (70%)	57 (30%)	
TSH (mIU/L)		1.97 (1.22–3.69)	2.1 (1.46–3.2)	0.09	2.3 (1.3–3.5)	1.8 (1.8–3.25)	0.03
Free T4 (ng/dl)		8.55 (6.75–11.42)	8.77 (7.51–10.15)	0.98	8.5 (6.5–9.8)	8.83 (7.8–10.1)	0.05
FSH (IU/L)		9.95 (7.92–17.55)	5.5 (4.1–6.8)	0.0001	8.7 (5.85–11.85)	6.5 (4.75–7.8)	0.0001
E2 (pg/ml)		39.5 (26.67–70.45)	48 (37.2–68.15)	0.12	57.1 (33–81.15)	49 (35.6–68.7)	0.29

*Mann–Whitney U test

*AMH* Anti-Mullerian hormone, *IQR* Interquartile range, *FSH* Follicle-stimulating hormone, *E2* Estradiol, *TSH* Thyroid stimulating hormone, *Free T4* Thyroxine

Results showed that despite the higher frequency of clinical hypothyroidism in the group with AMH < 1.1 ng/ml in both age groups, this difference was not statistically significant (*P*-value> 0.05) (Table 1). The partial correlation test was used to examine the relationship between AMH, and Free T4, FSH, TSH, and E2 variables in the whole cohort and the two sub-groups (AMH ≥ 1.1 ng/ml and AMH < 1.1 ng/ml) controlling and eliminating the effect of age (Table 2). Results showed significant correlation between AMH and FSH in the both groups with AMH < 1.1 ng/ml and AMH ≥1.1 ng/ml. It indicates that if age is under control and all patients are the same age, FSH is inversely related to AMH in both AMH groups. Also, the partial correlation coefficient’s between TSH and FSH in the group with AMH ≥ 1.1 ng/ml and group with AMH < 1.1 ng/ml shows that with age control there is a significant positive relationship between TSH and FSH in both AMH groups (*P*-value =0.001) (Table 2).

**Table 2:** The relationship between AMH and Free T4, FSH, TSH, E2 variables in the two sub-groups of participants based on AMH levels.

Variables		AMH	FSH	E2	TSH	FreeT4
All participants	AMH	1				
	FSH	−0.140*	1			
	E2	−0.06	− 0.061	1		
	TSH	−0.079	0.363**	−0.022	1	
	Free T4	− 0.009	−0.018	0.006	− 0.003	1
AMH < 1.1 ng/ml	AMH	1				
	FSH	−0.326**	1			
	E2	−0.009	− 0.102	1		
	TSH	−0.01	0.394**	−0.06	1	
	Free T4	− 0.092	−0.015	− 0.026	0.011	1
AMH ≥ 1.1 ng/ml	AMH	1				
	FSH	−0.108	1			
	E2	−0.054	− 0.032	1		
	TSH	−0.051	0.077	0.019	1	
	Free T4	0.044	−0.155	0.137	−0.077	1

Partial correlation test, *P-value< 0.05 ** *P*-value< 0.001

*FSH* Follicle-stimulating hormone (IU/L), *AMH* Anti-Mullerian hormone (ng/ml), *E2* Estradiol (pg/ml), *TSH* Thyroid stimulating hormone (mIU/ml)

In multivariate logistic regression, TSH and age variables were significantly associated with low AMH, so that with one unit increase in TSH level, the odds of having AMH < 1.1 ng/ml increases by 1.25 times or by 25% (*P*-value =0.017) (Table 3). Also, with an increase of 1 year in the age of patients, the odds of low AMH under the same conditions of variables increases by 1.296 times or 29.6% (*P*-value < 0.001) (Table 3). ROC curve analysis showed that TSH cut-off point of 1.465 mIU/L in participants over 35 years had 80.5% sensitivity, 38.6% specificity, and area under curve (AUC) = 0.596 in identifying decreased AMH level.

**Table 3:** Relative impact of demographic and clinical variables on ovarian reserve on regression model (backward model).

Factors affecting AMH	Regression coefficient’s	Standard error	(Adjusted Odd Ratios)	Confidence Interval 95%		*P*-value
				Lower limit	Upper limit	
TSH	0.223	0.093	1.250	1.500	1.041	0.017
Age	0.259	0.030	1.296	1.375	1.220	0.0001
Constant	−10.126	1.195	0.0001			0.0001

Multivariate logistic regression

## Discussion

Both thyroid disorders and decreased ovarian reserve increase with aging. Autoimmune thyroid disorders have also been reported in 10 to 30% of patients with ovarian failure, suggesting thyroid disorders are associated with ovarian reserve [14, 15]. In the present study, we examined the relationship between TSH and free T4 levels and markers of ovarian reserve, including AMH, E2, and FSH measured on the second day of the menstrual cycle. Seventy percent of the participants aged 35 years or older and 19.4% of the participants under the age of 35 years had AMH < 1.1 ng/ml (*P*-value < 0.001). This finding is in line with other studies showing that aging is associated with a decrease in the quality and quantity of ovarian function [16–19]. It has been reported that ovarian reserve depletion and its acceleration occur at the age of 37.5 years [20]. Surprisingly, 19.4% of women aged less than 35 years in our study had AMH level < 1.1 ng/ml. This may be due to the selection of participants among infertile women. The present study showed higher FSH levels in participants with AMH < 1.1 ng/ml in both age groups (*P*-value = 0.001). This result is consistent with other studies that showed a negative relationship between AMH and FSH, and it has been suggested that reduced ovarian function causes changes in both quantity and quality of ovarian factors (inhibin) and FSH is increased through the feedback system [21]. Our results demonstrated that, in participants aged 35 years or older median TSH was higher in participants with AMH < 1.1 ng/ml as compared to those with AMH ≥ 1.1 ng/ml (*P*-value = 0.037). In contrast, in younger participants below the age of 35 years, we were unable to demonstrate significantly different TSH level between the participants with AMH < 1.1 ng/ml and those with AMH ≥1.1 ng/ml (*P*-value = 0.09). Our findings were based on a single TSH measurement, and the failure to achieve statistically significant results on the relationship between TSH and ovarian reserve in younger participants can be due to physiological variability of TSH concentrations. According to our multiple logistic regression analysis (backward method), only the TSH and age remained significantly associated with AMH, so that with an increase of one unit in TSH values, the odds of patients being in the low AMH group at the same age increases 1.25 times or by 25% (*P*-value = .017). Also, with an increase of one unit in the age of patients, the chance of patients being in the low AMH group under the same conditions of TSH level increases 1.296 times or increases by 29.6% (*P*-value <.001). Our ROC curve analysis showed that a TSH cut-off point of 1.465 mIU/L in participants over 35 years predicted decreased AMH level with 80.5% sensitivity, 38.6% specificity, and AUC = 0.596.

Our finding contradicts a number of other studies [9, 10, 15]. In 2015 a cross-sectional analytical study [22] that was conducted in a large cohort population, all three groups of patients with normal, low, and high ovarian response were included. They found no relationship between thyroid hormone level and AMH level and their results were replicated by several other studies [13, 22–24]. However, most of these have been retrospective studies. Several studies have supported the relationship between TSH and AMH levels and have suggested that even in euthyroid patients, serum TSH levels less than 3 mIU/ml are associated with better ovarian function and if TSH is higher than this cut-off point, they recommended levothyroxine treatment [11, 25]. On the contrary, in Kuroda et al. (2018) study [26], treatment with levothyroxine failed to change ovarian reserve in clinical and subclinical hypothyroidism even though AMH level was significantly increased in patients with Hashimoto thyroiditis. Michalakis et al. (2011) have shown an association between TSH level and ovarian reserve, and in 18% of patients with low ovarian reserve, TSH levels were higher than 4 μIU/mL [9].

The strengths of our study include the prospective study design, the ability to select patients correctly, performing biochemical tests in a single laboratory and the assessment of several ovarian reserve markers. Also, we adjusted several factors that may affect ovarian reserve. Limitations of our study include the inability to evaluate anti-thyroid antibodies and the small sample size. However, it has been previously demonstrated that even after adjustment for thyroid autoimmunity and age, TSH < 3.0μIU/mL in euthyroid patients with infertility have significantly higher AMH level than as compared to those with TSH ≥3.0μIU/mL 14 [11]. Another limitation of the study was the selection of participants from infertile women population. Also, levothyroxine treatment followed by a re-evaluation of the ovarian reserve was not performed in this study.

## Conclusion

Our study supports the inverse relationship between thyroid-stimulating hormone and ovarian reserve, and an increase in TSH from a cut-off point of 1.465 mIU/L in participants over 35, is associated with a decrease in ovarian function. Larger prospective studies are needed to confirm these findings.

## Data Availability

Not applicable.
